# circINSR Promotes Proliferation and Reduces Apoptosis of Embryonic Myoblasts by Sponging miR-34a

**DOI:** 10.1016/j.omtn.2019.12.032

**Published:** 2020-01-14

**Authors:** Xuemei Shen, Xiaoyan Zhang, Wenxiu Ru, Yongzhen Huang, Xianyong Lan, Chuzhao Lei, Hong Chen

**Affiliations:** 1 Key Laboratory of Animal Genetics, Breeding and Reproduction of Shaanxi Province, College of Animal Science and Technology, Northwest A&F University, Yangling 712100, Shaanxi, China

**Keywords:** muscle development, bovine, circular RNAs, proliferation, miR-34a

## Abstract

As a diverse and abundant class of endogenous RNAs, circular RNAs (circRNAs) participate in processes including cell proliferation and apoptosis. Nevertheless, few researchers have investigated the function of circRNAs in bovine muscle development. Based on existing sequencing data, we identified circINSR. The localization of circINSR in bovine myoblasts was investigated by fluorescence *in situ* hybridization. Molecular and biochemical assays were used to confirm the role of circINSR in myoblast proliferation and the cell cycle. Mitochondrial membrane potential and annexin V-PE/7-AAD staining assays were performed to assess cell apoptosis. Additionally, interactions between circINSR, miR-34a, and target mRNAs were examined using bioinformatics, a luciferase assay, and RNA immunoprecipitation. We found that circINSR was highly expressed in embryonic muscle tissue. Overexpression of circINSR significantly promoted proliferation and reduced apoptosis of embryonic myoblasts. Our data suggested that circINSR may act as a sponge of miR-34a and could function through de-repression of target genes in muscle cells. This study proposes that circINSR may function as a regulator of embryonic muscle development. circINSR regulates cells proliferation and apoptosis through miR-34a-modulated Bcl-2 and CyclinE2 expression.

## Introduction

Skeletal muscle, as the largest motor organ of mammals, is currently the focus of research. For humans, muscle developmental diseases such as Duchenne muscular dystrophy (DMD) and facial muscular dystrophy (FSHD) threaten human health.^1^ For meat livestock, the development of skeletal muscle directly impacts the commercial value. Therefore, a deeper elucidation of the molecular mechanisms underlying muscle development would prove valuable for muscle diseases and cattle breeding. Muscle development is a complex process involving multiple genes and non-coding RNAs (ncRNAs), but the specific mechanisms remain to be explored.^2^

MicroRNAs (miRNAs) are a class of endogenous ncRNAs that inhibit the expression of downstream target genes through post-transcriptional regulation.^3^ There is increasing evidence that demonstrates miRNAs regulate a variety of biological processes, including muscle cell proliferation, differentiation, and apoptosis. For example, the elevation of serum levels of miRNAs (miR-1, miR-133, miR-206, miR-208a, miR-208b, and miR-499) was observed in DMD patients.^4^^,^^5^ miR-1, miR-206, and miR-486 promote muscle cell differentiation by inhibiting the transcription of Pax7,^6^^,^^7^ while miR-378a-3p promotes skeletal muscle cell differentiation by targeting HDAC4.^8^ Additionally, miR-27a targets myostatin and Pax3 to promote myocyte production and proper migration of myogenic progenitor cells.^9^^,^^10^ Moreover, miR-34 has been shown to be a direct transcriptional target of p53, which in turn downregulates the genes required for cell proliferation.^11^

Circular RNAs (circRNAs) are a class of endogenous ncRNAs whose covalent closed loop structure is formed by reverse splicing.^12^ This special structure makes circRNAs more stable than linear RNAs, which is beneficial for its physiological functions.^13^ With the development of RNA deep-sequencing technology, a large number of circRNAs have been found in a variety of cells. There is increasing evidence that circRNAs located in the cytoplasm can act as competing endogenous RNAs (ceRNAs) to countervail miRNAs and thus participate in a variety of physiological processes.^14^ circHIPK3 can sponge multiple miRNAs such as miR-7, miR-30a-3p, miR-124, miR-193a, and miR-558 and participate in the regulation of cell proliferation and migration of multiple cancer cell types.^15^, ^16^, ^17^, ^18^, ^19^ circYAP1 has a tumor suppressor effect and can inhibit the proliferation and migration of gastric cancer cells by targeting miR-367-5p.^20^ However, most research has focused on cancer medicine, with considerably fewer reports on the functions of circRNAs in animal muscle development. Previous research in our laboratory has shown that circFUT10 can act as a ceRNA to promote the expression of the SRF gene by competing with miR-133a, thereby inhibiting the proliferation of bovine myoblasts.^21^ circFGFR4 binds to miR-107, thereby releasing its inhibitory effect on the target gene Wnt3a and ultimately promoting cell differentiation.^22^ Despite these findings, further research is needed to better understand the roles of circRNAs in bovine muscle growth.

In our previous study, we identified hundreds of circRNAs that are differentially expressed in prenatal and adult development stages of bovine skeletal muscle.^8^ After analysis of these circRNAs, we further characterize a highly expressed circINSR in embryonic muscle tissue. circINSR is named after the insulin receptor (*INSR*) gene and is formed by cyclization of the second exon of *INSR*. *INSR* is highly expressed in insulin-targeted tissues, such as liver, adipose, and skeletal muscle. For muscle cells, INSR receives insulin signals, which promote the uptake of glycogen and accelerate protein synthesis.^23^ INSR is closely related to muscle nutrition and metabolic diseases during the embryonic period.^24^ Moreover, circINSR is highly homologous to human has_circ_0048966 (CircBase, http://www.circbase.org), which suggests that circINSR has important and potentially conserved functions.

In this study, we first explored the endogenous expression and functions of circINSR in muscle cells. We further clarified the possible regulatory relationships among circINSR, miR-34a, and target mRNAs. We found that circINSR promoted cell proliferation and inhibited cell apoptosis by sponging miR-34a in bovine myoblasts. These results provide potential molecular targets for improving beef cattle breeding and preventing muscle disease.

## Results

### Identification of circINSR as a Candidate circRNA

**Figure 1:**
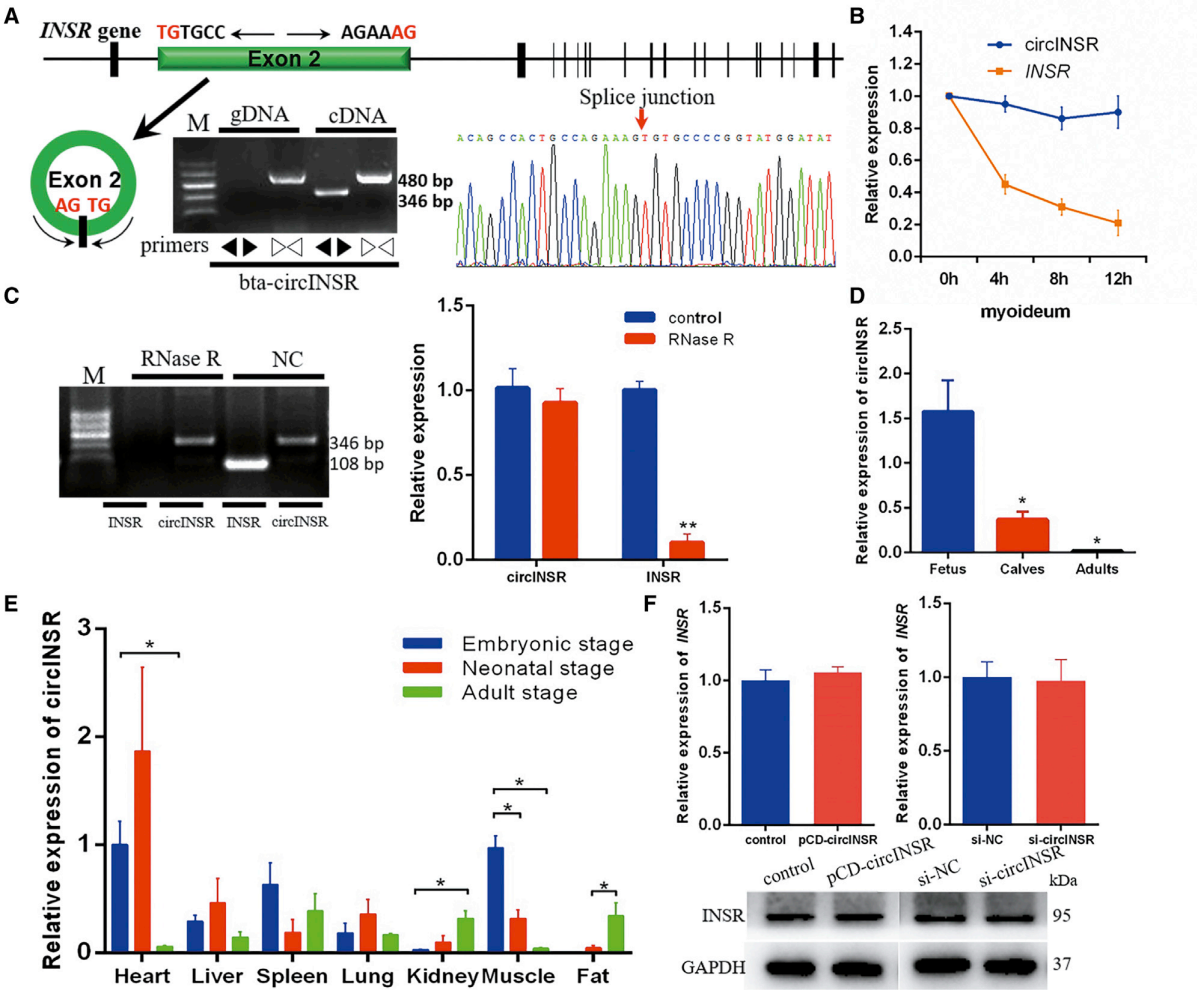
circINSR Identification and Expression Pattern in Bovine Skeletal Muscle (A) Schematic showing the circularization of *INSR* exon 2 forming circINSR (black arrow). The existence of circINSR was validated by agarose gel electrophoresis and followed by Sanger sequencing. Divergent primers amplified circINSR in cDNA but not genomic DNA (gDNA). Red arrow represents “head-to-tail” splicing sites of circINSR. (B) Myocytes were treated with the transcription inhibitor actinomycin D, and quantitative real-time PCR was used to detect the expression of circINSR and INSR mRNA at different time intervals. (C) Agarose gel electrophoresis and quantitative real-time PCR for the abundance of circINSR with or without RNase R treatment. *INSR* was used as a negative control. (D) The expression of circINSR in muscle tissues of cattle at three developmental stages. (E) The expression of circINSR in different tissues of cattle at three developmental stages. Data are presented as means ± SEM. *p < 0.05. (F) The expression of the *INSR* gene was detected using quantitative real-time PCR and western blots after overexpression and interference with circINSR.

To better reveal the role of circRNAs in muscle development, we screened differentially expressed circINSR in our published sequencing data (NCBI: GSE87908). According to the online database CircBase (http://www.circbase.org), we found that circINSR is highly homologous to human has_circ_0048966, both of which consist of head-to-tail splicing of *INSR* exon 2 (552 bp). circINSR was only amplified in cDNA by divergent primers, and no amplification product was observed in genomic DNA (gDNA). The amplified product of circINSR was confirmed by sequencing technology (Figure 1A). Actinomycin D inhibits mRNA synthesis and promotes RNA degradation. After treatment with actinomycin D, the expression of circINSR in bovine myoblasts was slightly reduced. However, the expression of INSR mRNA was greatly reduced in a time-dependent manner (Figure 1B), and the difference in half-life between circINSR and INSR mRNA reflected the stability of circINSR. Moreover, circINSR was resistant to RNase R treatment compared to linear mRNA (Figure 1C).

Analysis of tissue expression patterns showed that the expression of circINSR in embryonic muscle was significantly higher than that in adulthood (Figure 1D). This result is consistent with the sequencing data. Quantitative assays revealed that circINSR was expressed in several bovine tissues, including heart, liver, spleen, lung, kidney, and subcutaneous adipose tissues. It reveals the increasing trend of circINSR during the development of individuals in the kidney and adipose tissue, while in the muscle tissue, changes are opposite. Expression levels in embryonic myocardium and muscle were significantly higher than in other tissues (Figure 1E). circRNA may regulate different stages of gene expression; also, they often affect the expression of genes within which they are encoded.^25^ We analyzed the expression of the *INSR* gene after overexpression and interference with circINSR. It was determined that the levels of *INSR* gene and protein were not associated with circINSR, and the subsequent test results were not affected by the INSR gene (Figure 1F).

### Overexpression of circINSR Promotes the Myocytes’ Proliferation

**Figure 2:**
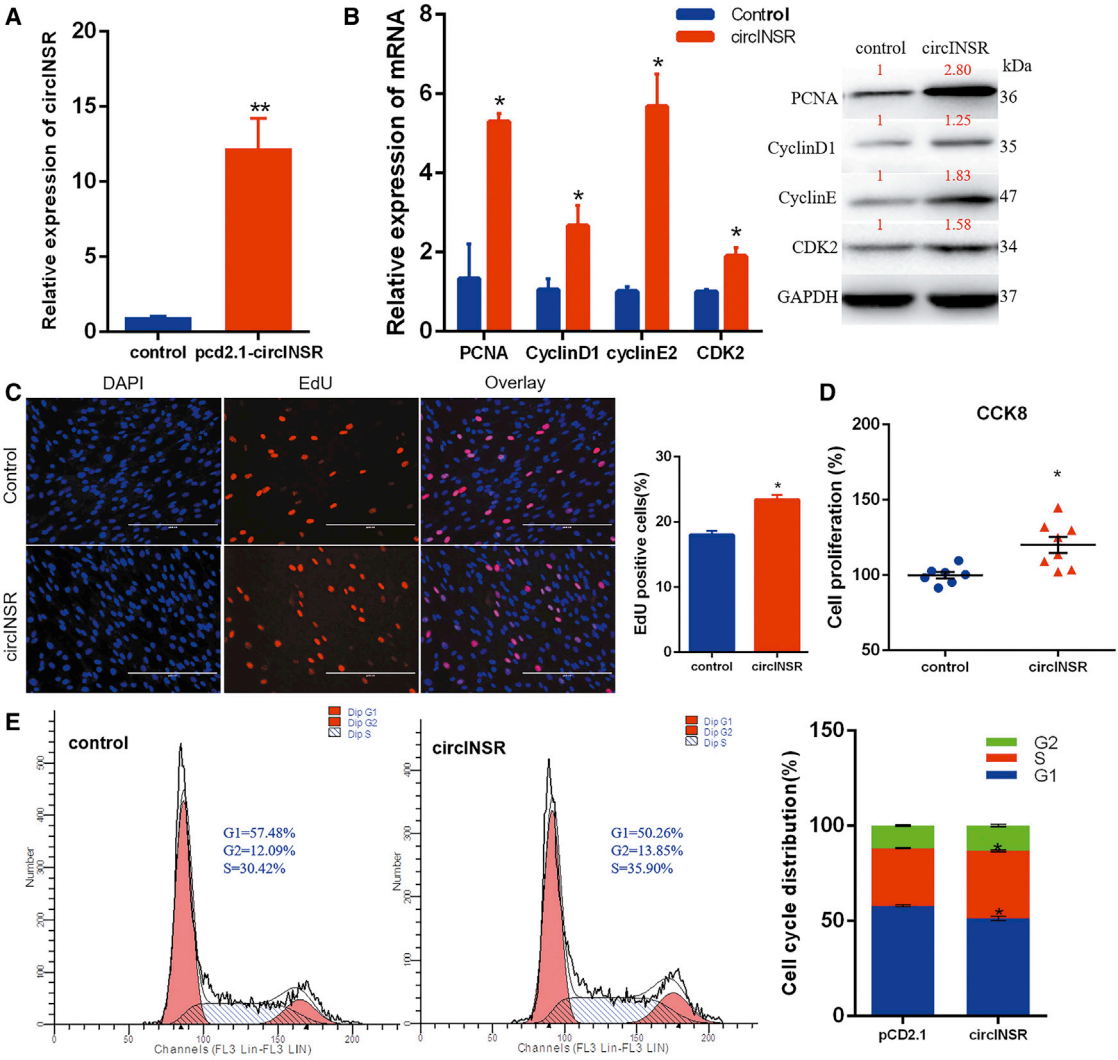
Overexpression of circINSR Promotes the Proliferation of Bovine Primary Myocytes *In Vitro* (A) The overexpression transfection efficiency of circINSR. (B) The expression of PCNA, CyclinD1, CyclinE, and CDK2 was detected by quantitative real-time PCR and western blots. These red numbers were the relative grayscale values of the proteins. (C) Bovine primary myocytes were transfected with pCD2.1-circINSR, and cell proliferation was analyzed using EdU (scale bars, 2,000 μm) and counted using a public domain software Image J (NIH Image, Bethesda, MD). EdU staining (red) for positive cells. Hoechst 33342 staining (blue) for the cell nuclei. (D) Cell proliferation index was detected by cell counting kit-8 (CCK-8) assay. (E) The cell cycle phase index was analyzed by flow cytometry. Date are presented as means ± SEM for three individuals. *p < 0.05, **p < 0.01.

To explore the function of circINSR, the overexpression vector of the pCD2.1-circINSR plasmid was transfected into myoblasts. Overexpression of plasmid led to a >10-fold induction of circINSR (Figure 2A) and significantly increased the expression of proliferation-related genes, including PCNA, CyclinD1, CyclinE2, and CDK2, at both mRNA and protein levels (Figure 2B). 5-ethynyl-2′-deoxyuridine (EdU) staining analysis indicated that overexpression of circINSR significantly increased the number of myoblasts in the proliferative phase. Cell counting kit-8 (CCK8) detection showed the same results (Figures 2C and 2D). In order to investigate whether circINSR affects the cell cycle, we used flow cytometry to analyze the phase distribution of proliferating myoblasts. These results indicated that the number of cells at G1 decreased while those at the S phase increased upon circINSR overexpression (Figure 2E). The cell cycle change in G2 phase was not significant. The results also indicated that circINSR released the G1 phase arrest and promoted myocyte proliferation.

### Silencing of circINSR Inhibits Myocyte Proliferation

**Figure 3:**
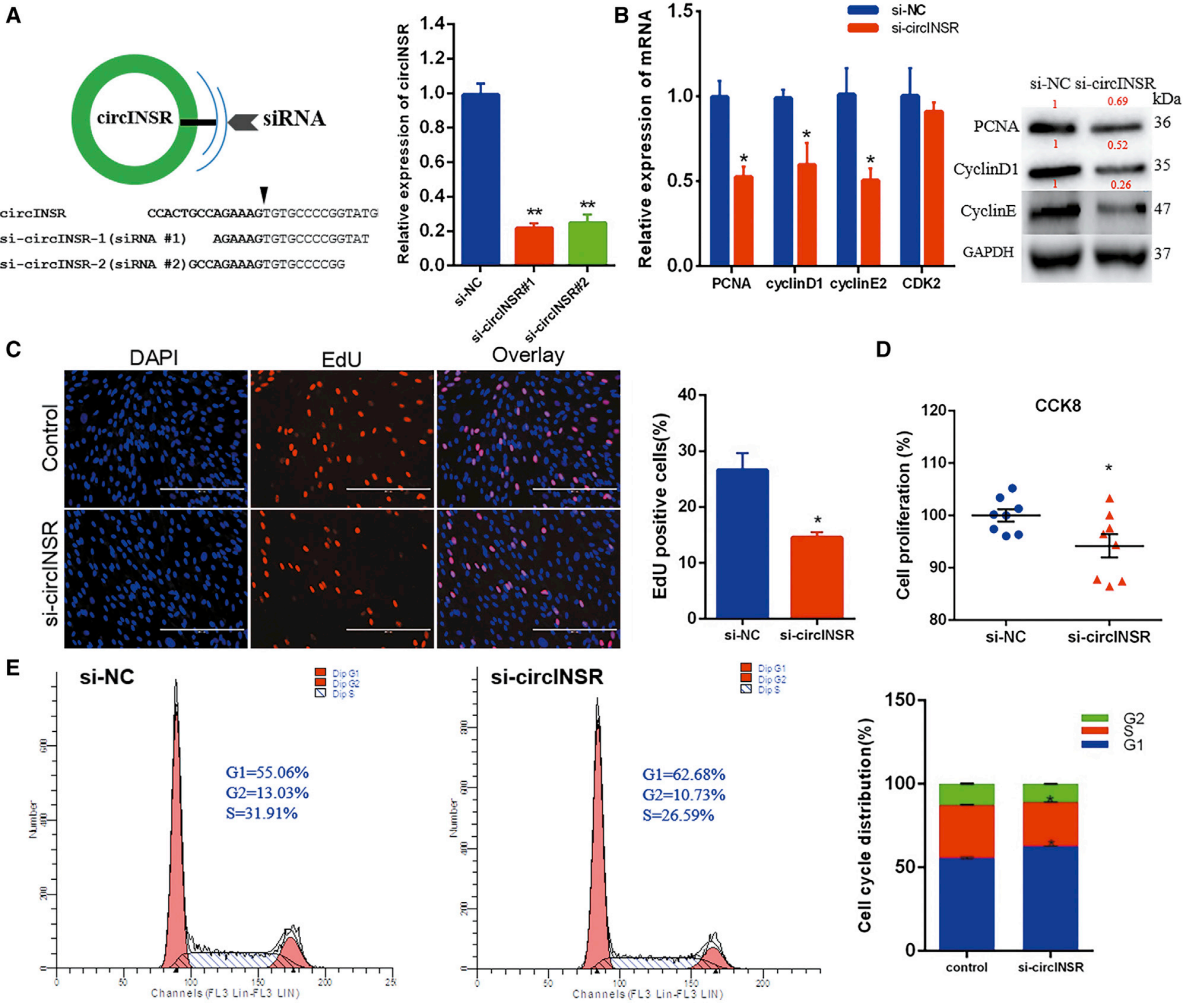
Interfering with circINSR Inhibits the Proliferation of Bovine Primary Myocytes *In Vitro* (A) Myocytes were transfected with siRNA#1, siRNA#2, or scrambled (NC) siRNA for 24 h. The quantitative real-time PCR assays were conducted to detect circINSR expression (n = 3, **p < 0.01 versus NC group). (B) The expression of PCNA, CyclinD1, CyclinE, and CDK2 was detected by quantitative real-time PCR and western blots. (C and D) Bovine primary myocytes were transfected with pCD2.1-circINSR, and cell proliferation was analyzed using EdU (scale bars, 2,000 μm) (C) and cell counting kit-8 (CCK8) (D). (E) The cell cycle phase index was analyzed by flow cytometry. Date are presented as means ± SEM for three individuals. *p < 0.05.

**Figure 4:**
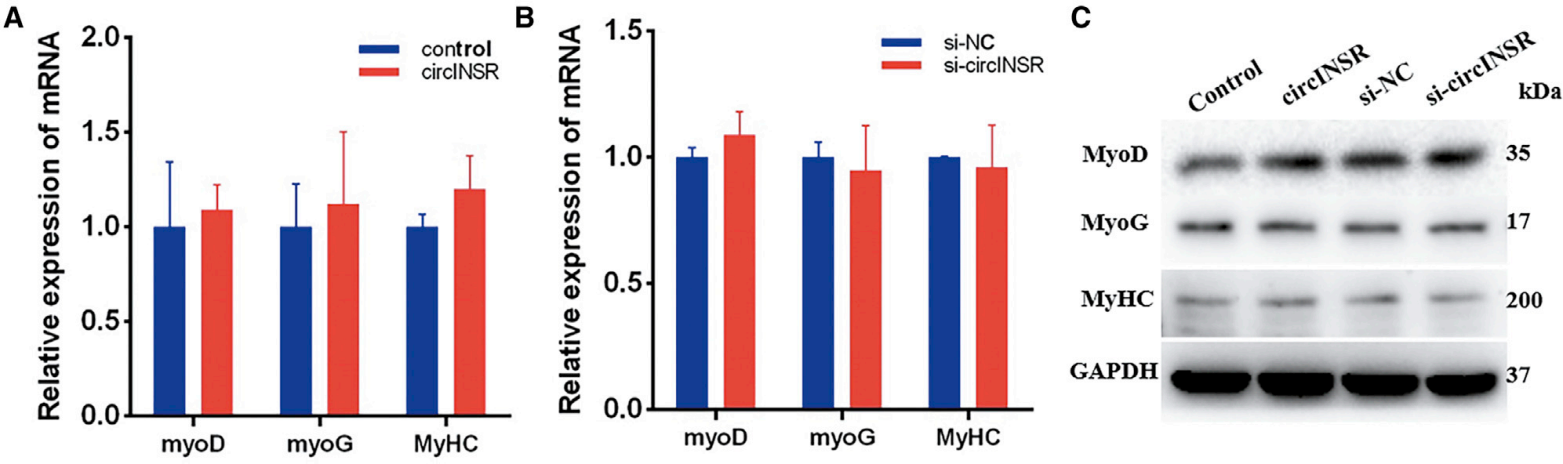
Overexpression and Interference with circINSR Had No Effect on Primary Muscle Cell Differentiation (A) Bovine primary myocytes were transfected with pCD2.1-circINSR, and the expression of MyoD, MyoG, and MyHC was detected by quantitative real-time PCR. (B) The mRNA expression of cell differentiation markers was detected by quantitative real-time PCR. (C) The protein expression of MyoD, MyoG, and MyHC was analyzed by western blotting. Values are means ± SEM for three individuals.

We asked whether circINSR inhibition would produce an opposite effect of that which was observed by overexpression. Two different small interfering RNAs (siRNAs) were designed for circINSR silencing. Both siRNA#1 and siRNA#2 directed against the back-splice sequence knocked down the circular transcript (Figure 3A), and we randomly selected siRNA#1 for subsequent experiments. As expected, western blot and quantitative real-time PCR assays revealed that circINSR silencing significantly inhibited the expression of PCNA, CyclinD1, and CyclinE2 of bovine primary myocytes (Figure 3B). Cell proliferation was then determined by EdU and CCK8 assays, and cell viability in the si-circINSR group was significantly decreased in comparison with that in the control group (Figures 3C and 3D). Cell cycle analysis demonstrated that circINSR interference prevented normal cell cycle progression, resulting in more cells arresting in the G0/G1 phase and thus fewer cells in S and G2 stages (Figure 3E). These results demonstrate that circINSR interference inhibited the proliferation of muscle cells. Next, we examined the function of circINSR in muscle cell differentiation. Bovine primary myocytes were transfected with pCD2.1-circINSR, and the expression of MyoD (myogenic differentiation antigen), MyoG (myogenin), and MyHC (myosin heavy chains) were detected by quantitative real-time PCR. The results showed that circINSR might not participate in the differentiation of bovine primary myoblasts (Figure 4).

### Effects of circINSR on Cell Apoptosis

**Figure 5:**
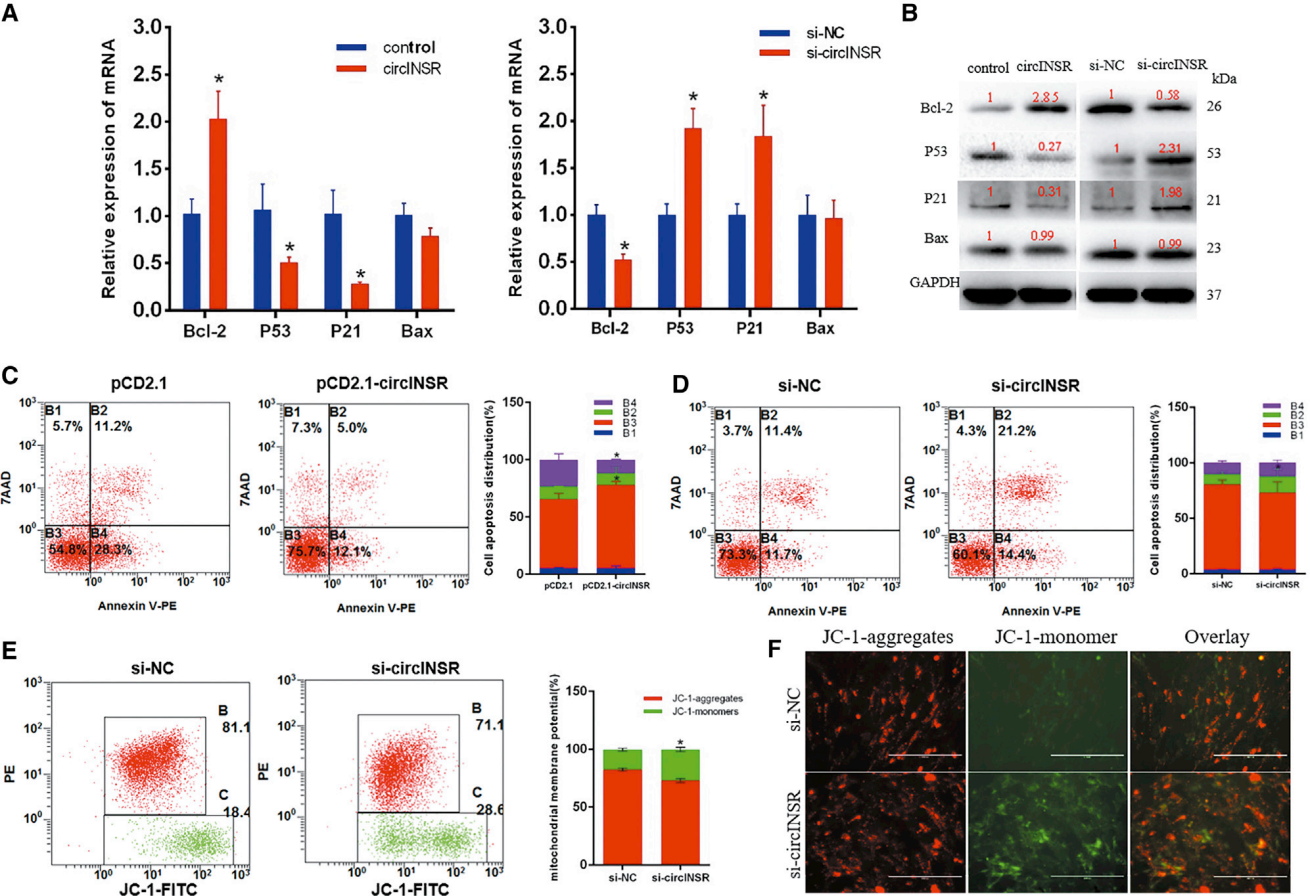
Effects of circINSR on Cell Apoptosis (A and B) The expression of apoptosis marker genes was detected using quantitative real-time PCR (A) and western blots (B). (C) After the cells overexpressed circINSR, apoptosis was determined by annexin V-PE/7-AAD dual staining followed by flow cytometry. (D) After terference with circINSR, apoptosis was determined by annexin V-PE/7-AAD dual staining followed by flow cytometry. (E) Treated cells were stained with JC-1 solution and then observed by flow cytometry. (F) Cells were stained with JC-1, and images were acquired using a fluorescence microscope. Scale bars, 2,000 μm. Data are shown as means ± SEM for three individuals. *p < 0.05.

To investigate whether circINSR regulates myoblast apoptosis, we used the quantitative real-time PCR and western blotting assays after overexpression or silencing of circINSR. The results showed that overexpression of circINSR can increase the expression of Bcl-2 and inhibit that of p53 and p21 but has no effect on Bax (Figures 5A and 5B). In view of the fact that the pCD2.1 vector has green fluorescence, we used annexin V-phycoerythrin/7-amino-actinomycin (annexin V-PE/7-AAD) staining instead of the commonly used annexin V-fluorescein isothiocyanate (FITC)/propidium iodide (PI) to measure apoptosis by flow cytometry. Introduction of siRNA against circINSR resulted in a significant accumulation of apoptotic cells, while the amount of overexpression decreased (Figures 5C and 5D).

Decreased mitochondrial membrane potential (ΔΨm) is a landmark event in the early stages of apoptosis. 5,5′,6,6′-tetrachloro-1,1′,3,3′-tetraethyl-imidacarbocyanine iodide (JC-1) is an ideal fluorescent probe widely used to detect ΔΨm. The transition from red fluorescence to green fluorescence of JC-1 can be used as a marker for early detection of apoptosis. To minimize interference from pCD2.1 vector green fluorescence on the JC-1 fluorescence signal, we only detected the ΔΨm after interference. After JC-1 staining, we detected red and green fluorescence in the cells by flow cytometry and fluorescence microscopy, respectively. Compared to the untreated group, si-circINSR treatment resulted in a remarkable increase in green fluorescence, revealing that circINSR interference could induce mitochondrial membrane depolarization (Figures 5E and 5F). These results indicated that circINSR might inhibit apoptosis via the mitochondrial apoptosis pathway.

### circINSR Acts as a Molecular Sponge for miR-34a

**Figure 6:**
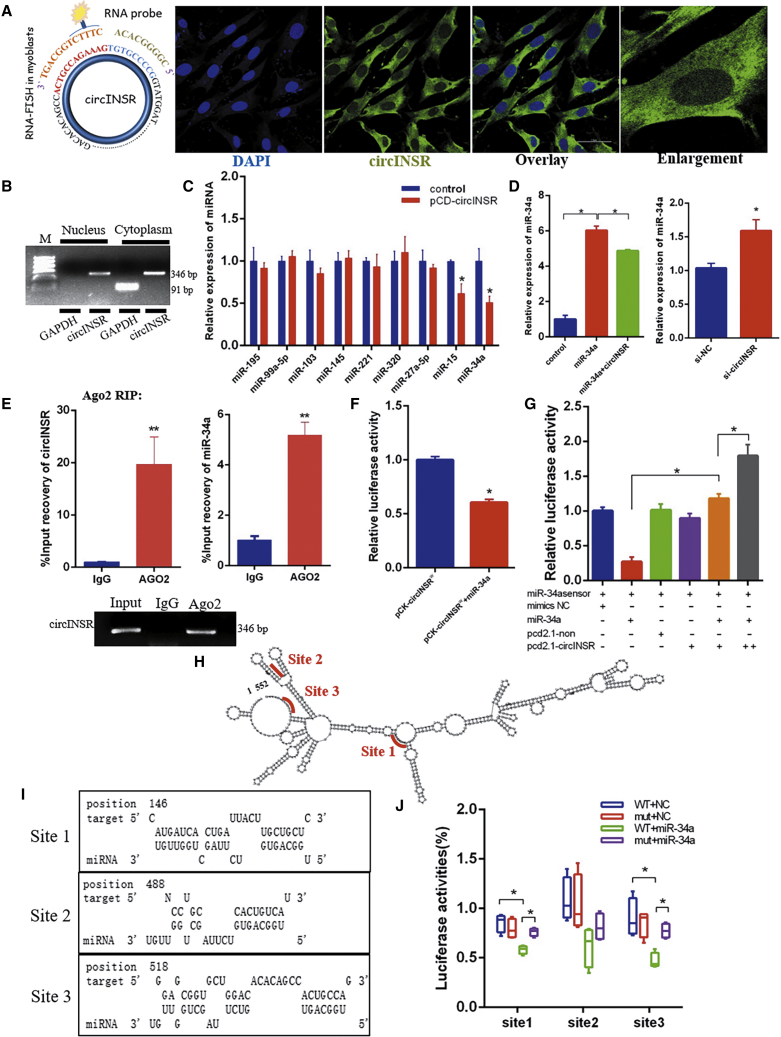
circINSR Serves as a miR-34a Sponge *In Vitro* (A) RNA-FISH showed that circINSR is mainly localized in the cytoplasm of myoblasts. Blue indicates nuclei stained with DAPI; green indicates the RNA probe that recognizes circINSR. Scale bars, 50 μm. (B) Distribution of circINSR after separation of nucleus and cytoplasm. (C) Effect of circINSR on the abundance of miRNAs. (D) Effects of overexpression and interference with circINSR on the expression level of miR-34a. (E) Ago2-RIP assay for the amount of circINSR and miR-34a in bovine myoblasts. RIP experiments showed that the anti-AGO2 antibody efficiently captured circINSR and miR-34a transcripts. **p < 0.01. (F) miR-34a mimics were co-transfected with psiCHECK2-circINSR-WT (pCK-circINSR^W^) into HEK293T cells. Renilla luciferase activity was normalized to firefly luciferase activity. (G) The miR-34a biosensor (psiCHECK2-miR-34a 2×) was transfected into HEK293T cells, together with mimics-NC, miR-34a mimics, pCD-2.1-non, or pCD2.1-circINSR, and luciferase activities were measured after transfection. (H) The predicted secondary structure of circINSR and the absorption site information. (I) The predicted miR-34a binding sites at three distinct positions in circINSR. (J) HEK293T cells were transfected with miR-34a and luciferase constructs of circINSR containing wild-type putative miR-34a binding sites (circINSR site WT) or mutated sites (circINSR site mut). n = 6; *p < 0.05.

We designed an RNA probe that specifically recognizes the back-splicing junction region of circINSR. RNA-fluorescence *in situ* hybridization (FISH) localization and PCR results indicated that circINSR was mainly localized in the cytoplasm of bovine myoblasts (Figures 6A and 6B), so we assume that circINSR acts as a miRNA sponge. According to the results described above, circINSR could inhibit the expression of p53 and promote cell proliferation. To find miRNAs that interact with circINSR, we examined a number of miRNAs involved in the p53 pathway and cell proliferation processes after overexpression of circINSR (Figure 6C). We found that the expression of miR-34a and miR-15 was decreased after overexpression of circINSR, while the expression of miR-34a was increased after interference with circINSR (Figure 6D). Several studies have found that members of the conserved miR-34 family are involved in the p53 network and that the miR-34 family may have roles in tumor cell proliferation and migration.^11^ Given that circRNAs function by adsorbing miRNAs as molecular sponges, we next investigated the ability of circINSR to target miR-34a.

To validate this adsorption, we conducted an Ago2-RNA-binding protein immunoprecipitation (RIP) assay in bovine myoblasts and examined the expression of endogenous circINSR and miR-34a bound to Ago2 protein. The results indicated that circINSR and miR-34a were highly enriched in the Ago2 pellet (Figure 6E). The luciferase activity of the psiCHECK2-circINSR-wild type (pCK-circINSR^w^)+miR-34a group was significantly lower than the pCK-circINSR^w^ group (Figure 6F). The addition of a repeat motif complementary to the miR-34a mature sequence in the psiCHECK-2 vector became a strong positive control for the dual fluorescence assay. Accordingly, a miR-34a biosensor was transfected into HEK293T cells, together with sponge plasmid pCD2.1-circINSR, pCD2.1-non (vector), miR-34a mimics, or mimics-negative control (mimics-NC). miR-34a overexpression inhibited the luciferase expression of the biosensor, and when miR-34a and circINSR overexpression vectors were co-transfected, circINSR adsorbed miR-34a, thereby alleviating the inhibitory effect of miR-34a on the fluorescence of the biosensor, the recovery of which exhibited a dose-dependent effect (Figure 6G). The three binding sites for miR-34a on circINSR were predicted using TargetScan 7.0 and miRanda (Figures 6H and 6I). Due to more than one miR-34a adsorption site on circINSR, we were unable to construct the mutant (MUT) vector. We synthesized the wild-type (WT) and MUT sequences at each site of the psiCHECK-2 to verify the expression of luciferase. Combining the results of luciferase screening assay with the predicted secondary structure, circINSR was observed to sponge miR-34a with two potential binding sites, although site 2 did not bind efficiently (Figure 6J).

### circINSR Regulated Cell Proliferation and Apoptosis in a miR-34a-Dependent Manner

**Figure 7:**
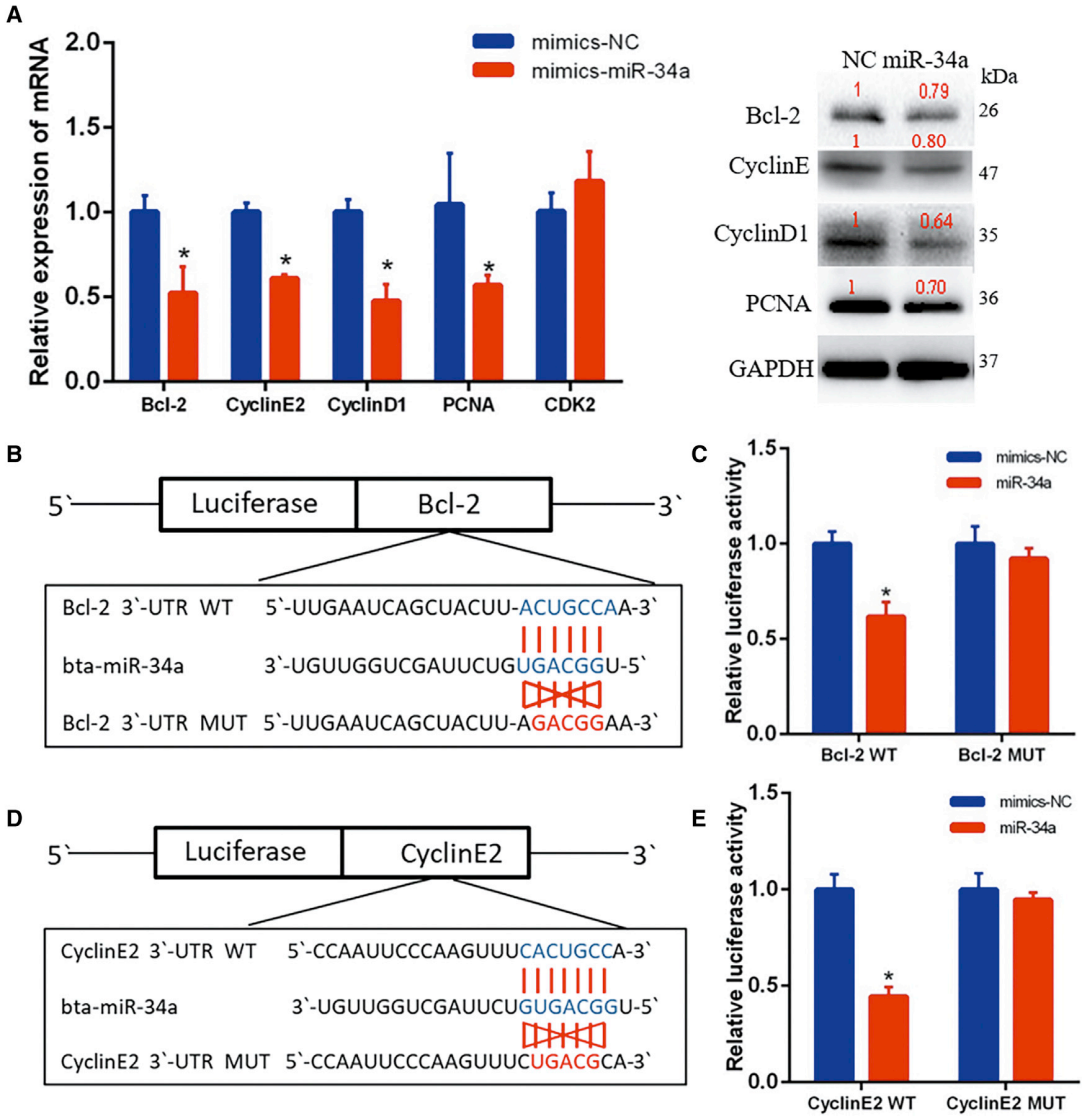
miR-34a Directly Targets *Bcl-2* and *CyclinE2* Genes (A) The expression of Bcl-2, CyclinE2, CyclinD1, PCNA, and CDK2 was detected by quantitative real-time PCR and western blots. (B and C) TargetScan predicted that Bcl-2 3′UTR had a binding site for miR-34a. The luciferase reporter assay was used to analyze the target relationship between Bcl-2 and miR-34a. (D and E) TargetScan predicted that CyclinE2 3′UTR had a binding site for miR-34a. The luciferase reporter assay was used to analyze the target relationship between CyclinE2 and miR-34a. Date are presented as means ± SEM for three individuals. *p < 0.05.

**Figure 8:**
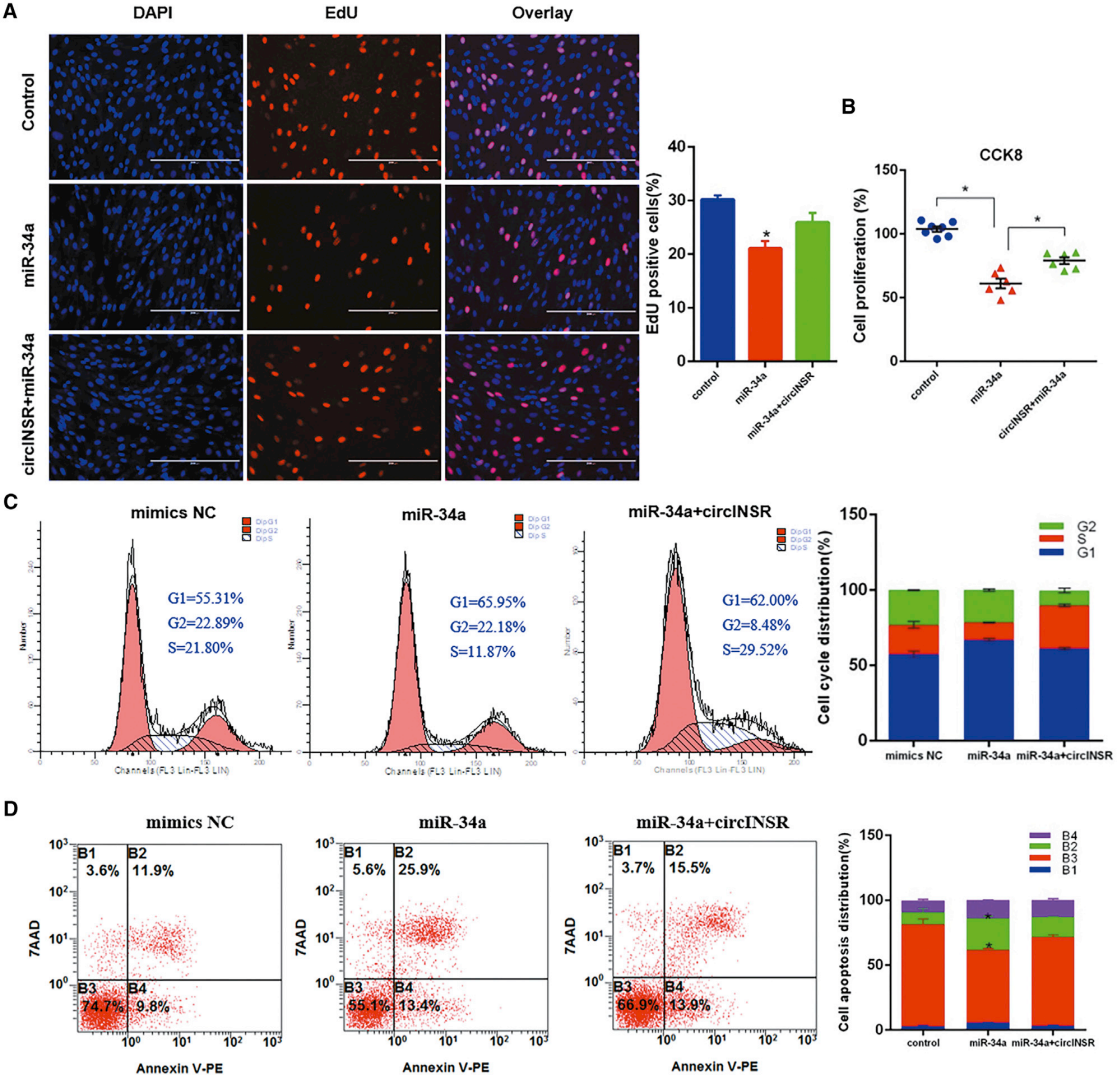
circINSR Regulated Cell Proliferation and Apoptosis in a miR-34a-Dependent Manner (A and B) Bovine primary myocytes were co-transfected with miR-34a and pCD2.1-circINSR, and cell proliferation was analyzed using EdU (A) and CCK8 (B). (C) The cell cycle phase index was analyzed by flow cytometry. (D) Cell apoptosis after co-transfection was determined by annexin V-PE/7-AAD dual staining followed by flow cytometry. Date are presented as means ± SEM for three individuals. *p <0.05 compared with the NC group.

Based on the results of previous studies, miR-34a could inhibit cell proliferation by inhibiting its target genes (*Bcl-2*, *MYC*, *CyclinD1*, *CyclinE2*, *NOTCH1*, *CDK4*, and *CDK6*),^11^^,^^26^ while the results of this study also indicated that overexpression of miR-34a can reduce the expression of target genes (*Bcl-2*, *CyclinD1*, and *CyclinE2*) and the proliferative marker gene *PCNA* (Figure 7A). As predicted by bioinformatics programs, Bcl-2, a well-known regulator of the apoptotic pathway, is a potential target of miR-34a (Figure 7B). CyclinE2 is an important member of the cyclin family. The luciferase reporter assay showed that miR-34a mimics significantly inhibited the relative luciferase activity of Bcl-2 WT and CyclinE2 WT, but did not repress the mutant group (Figures 7C and 7E). Moreover, after co-transfection of miR-34a and circINSR, the results of EdU and CCK8 showed that the presence of circINSR can remove the inhibition of miR-34a on target genes, thus promoting cell proliferation (Figures 8A and 8B). A subsequent cell cycle assay indicated that overexpression of miR-34a significantly suppressed cell cycle progression. However, co-transfection of circINSR and miR-34a could reverse the inhibition of miR-34a on the cell cycle, consistent with the EdU and CCK8 results (Figure 8C). Previous studies have shown that circRNAs regulate cell growth and apoptosis in multiple types. In our study, bovine primary myoblasts were co-transfected with circINSR and miR-34a, and cell apoptosis was then analyzed. From the flow cytometry assay in Figure 8D, overexpression of miR-34a increased the number of apoptotic cells, while circINSR could inhibit the occurrence of apoptosis.

## Discussion

Initially, circRNAs were identified as byproducts of spliceosome-mediated splicing errors.^27^ In recent years, circRNAs have received extensive attention because of their involvement in various cellular physiological processes and critical roles in epigenetic regulation.^28^ The complexity of the regulatory functions of circRNAs provides us with more biomarkers associated with muscle diseases and animal breeding. Based on sequencing technology and biotechnology, an increasing number of circRNAs have been identified to be involved in mediating cell proliferation, differentiation, apoptosis, and autophagy. However, the functions of many circRNAs remain unclear.

During the embryonic period, muscle development is manifested by an increase in the number of fibers (hyperplasia). In adulthood, muscle fiber volume increases (hypertrophy), but the number of fibers does not change.^2^^,^^29^ For meat livestock, the embryonic period is a critical period in determining their economic value. Besides, the embryonic muscle development is an important indicator for detecting muscle diseases in therapeutic intervention. For example, DMD is a congenital disease with a high proportion in newborns. In early childhood, the skeletal muscles will severely degenerate and eventually extend to atrophy of body muscle.^5^ The newly discovered circINSR identified from bovine muscle sequencing is highly homologous to human circ-004869 but is not detected in mice. To date, this circRNA has not been reported on. Our sequencing results showed that the expression level of circINSR in embryonic muscle tissue was significantly higher than in adult muscle, which is consistent with the results of this study (Figure 1D). This suggests that the highly expressed circRNA in the embryonic muscle tissue may be involved in the regulation of muscle development.

In addition, circINSR is derived from the glycolipid utilization-related insulin receptor gene. In mammals, the *INSR* gene has divided into two subtypes due to the presence or absence of exon 11 (exon 11 minus *INSR-A* and exon 11 plus *INSR-B*).^30^ *INSR-A* is mainly expressed in the embryonic stage and, after stimulation with insulin-like growth factor-2 (IGF-2), promotes uptake of glucose and accelerates protein synthesis in muscle cells.^23^^,^^24^^,^^31^ In contrast, *INSR-B* is primarily expressed in the adult well-differentiated tissues, including the liver, where it enhances the insulin sensitivity. Hence, we speculated that circINSR plays a vital role in muscle development during the embryonic period.

Based on these speculations, we used overexpression and interference to explore whether circINSR has important functions in bovine muscle cell development. PCD2.1 is a vector that utilizes a reverse complement sequence to effect automatic circularization of the inserted sequence. The vector carries green fluorescent protein (GFP), which can be used to directly determine transfection efficiency. In this study, circINSR overexpression was achieved more than 10-fold compared to the control group (Figure 2A). In addition, siRNA was designed at the junction of circINSR and did not appear to interfere with any other linear RNA. The results of quantitative real-time PCR and western blot showed that circINSR could significantly increase the expression of cell proliferation-related proteins such as PCNA, CyclinD1, CyclinE, and CDK2. Cell proliferation and cycle phase distribution assays revealed that circINSR promotes cell proliferation and cell cycle progression (Figures 2 and 3). Subsequent experiments studied the effect of circINSR on apoptosis by quantitative real-time PCR, annexin V-PE/7-AAD staining, and ΔΨm detection. The results showed that circINSR could inhibit apoptosis of myocytes (Figure 5). All these results suggested that circINSR promoted myoblast proliferation and reduced apoptosis. However, the molecular mechanisms underlying these phenomena are still unclear.

Accumulating data show that circRNAs, composed of exons and predominantly localized in the cytoplasm, can act as miRNA sponges and induce the dysregulation of miRNA and their target genes.^32^ Searches for circRNAs regulating cell proliferation and apoptosis were previously attempted by binding miRNAs in various cells. Existing reports indicate that circHIPK3, which originated from exon 2 of the *HIPK3* gene and localized in the cytoplasm, was involved in the regulation of proliferation and migration of various cancer cells via sponging miRNAs.^16^, ^17^, ^18^, ^19^ circVMA21 was derived from the third exon of *VMA21* gene, located in the cytoplasm, and acted as a sponge of miR-200c to alleviate cell apoptosis.^33^ circDiaph3 was derived from the exon of diaphanous-related formin 3 (*Diaph3*, also known as MDIA2) and was the ceRNA of miR-148a-5p.^34^ In our study, RNA-FISH localization indicated that circINSR was expressed in the cytoplasm. In addition, circINSR could inhibit the expression of p53/p21 and promote the expression of Bcl-2, CyclinD1, and CyclinE2. After identifying all miRNAs associated with these genes, we focused on miR-34a.

**Figure 9:**
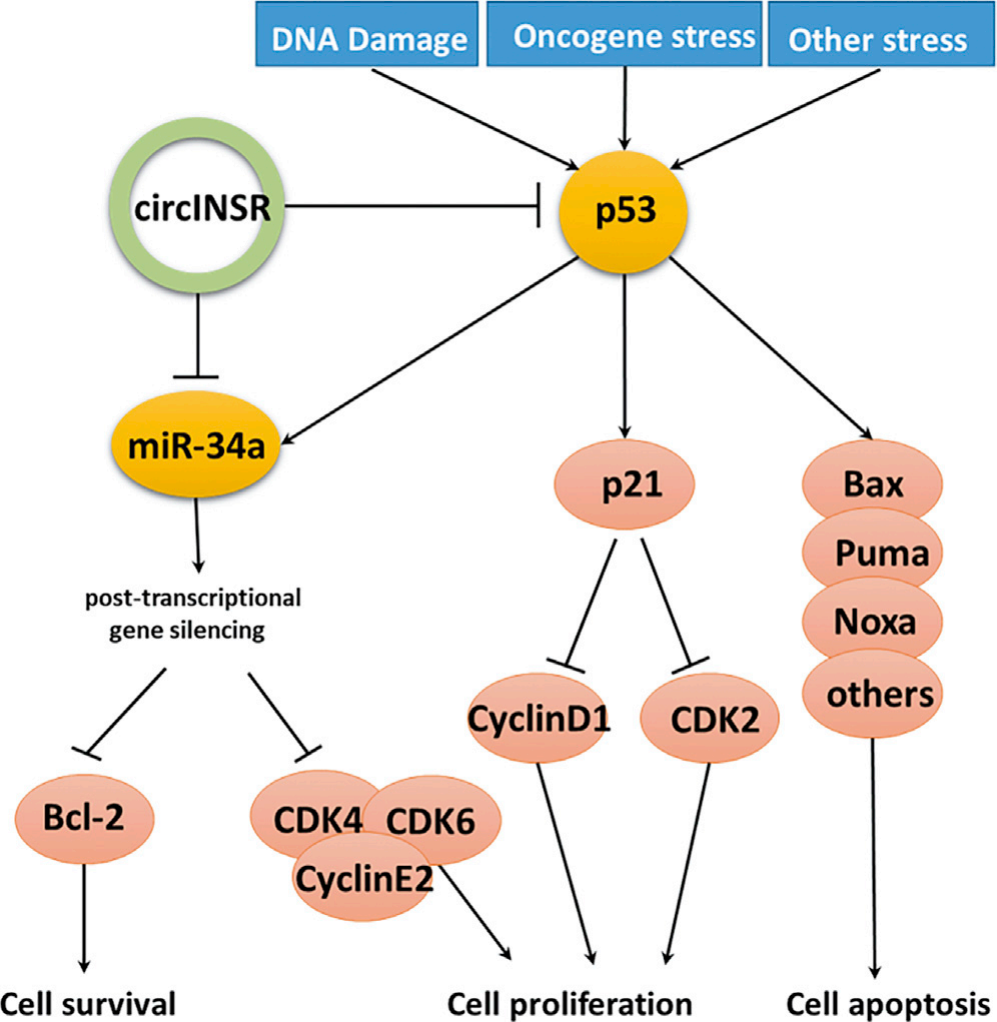
Schematic Diagram of circINSR Competitively Binding miR-34a in Regulating Cell Proliferation and Cell Apoptosis

In this study, we found that circINSR functions as a miR-34a sponge. Bioinformatics analysis revealed that circINSR contained two target sites of miR-34a, which were validated by luciferase and RIP analyses. miR-34a inhibits muscle cell proliferation and promotes apoptosis, but circINSR co-transfection with miR-34a could reverse this effect. In addition, the expression of miR-34a target mRNAs, Bcl-2, and CyclinE2 were positively modulated by circINSR (Figures 2B and 5A). Generally, Bcl-2 inhibits apoptosis by interfering with the aggregation of pro-apoptotic members, regulating the ΔΨm, and blocking the release of cytochrome *c* and other mediators,^35^^,^^36^ whereas p53 enhances apoptosis.^37^ The CyclinE2/CDK2 complex is the key kinase-mediating cell entry into S phase from G0/G1 phase.^38^ The CyclinE family of proteins can function by phosphorylating downstream substrates, such as CDC6, NPAT, and P107, thus enabling the cell to initiate DNA synthesis.^39^^,^^40^ Many studies have elucidated the important role of miR-34a in inhibiting cell proliferation. Therefore, it is often considered to be a tumor suppressor gene.^26^^,^^41^ For instance, Welch et al.^42^ demonstrated that miR-34a was a potential tumor suppressor that inhibits cell proliferation by targeting E2F3 transcription factors. As bona fide transcriptional targets of p53, the miR-34 family participated in the formation of a p53/miR-34/HDM4 feedback loop, which has a role in inducing the cell cycle and promoting apoptosis.^43^ These findings were in consistence with our findings (Figure 9).

Moreover, in kidney and adipose tissue, the expression of circINSR in adulthood was significantly higher than that in the neonatal period, which was contrary to the trend of muscle tissue (Figure 1E). This is possibly a significant example that the expression of circRNAs is regulated by development and is tissue and cell type specific. In addition, due to the special function of circINSR, other miRNAs may be adsorbed to regulate other cellular pathways. For example, circINSR, the focus of the present study, can also adsorb the miR-15/16 family to affect lipid deposition in adipocytes (unpublished data).

For the purposes of this study, circINSR could promote bovine embryonic muscle development by targeting miR-34a. From the perspective of the livestock industry, our findings extend the understanding of ncRNA related to bovine muscle development. circINSR could be used as a molecular marker for selection and breeding. For medicine, this circINSR is highly conserved in humans, and the function of circINSR is to regulate proliferation and apoptosis, so it could be useful as a molecular marker for muscle-related diseases, cancers, and other disease diagnoses. In summary, our study highlights the positive effect of circINSR on bovine muscle development, including increased cell proliferation, inhibition of cell cycle arrest, and suppressed cell apoptosis through targeting of miR-34a. However, it is still unknown whether this circRNA can regulate other miRNAs. Future research efforts should also be made to interrogate the function of circINSR in the nucleus.

## Materials and Methods

### Tissue Specimens

We collected a variety of tissue samples of Qinchuan cattle (*Bos taurus* Qinchuanensis) from three different growth stages each (three fetal [90-day] samples, three calf [1 month after birth] samples, and three adult [24-month] samples) from a local livestock farm in Xi’an, China. All specimens were immediately snap-frozen in liquid nitrogen after surgical removal. Animal care and study protocols were approved by the Animal Care Commission of the College of Veterinary Medicine, Northwest A&F University.

### Cell Culture and Treatment

As previously described, myoblasts were obtained from the bovine longissimus dorsi by collagenase digestion.^44^ Cell culture was performed using high-glucose Dulbecco’s modified Eagle’s medium (DMEM) supplemented with 20% fetal bovine serum (FBS, Gibco) at 37°C in a humidified incubator containing 5% CO_2_. HEK293T cells were cultured in DMEM containing 10% FBS. In order to induce primary myocyte differentiation, after the addition treatment, the complete medium was changed to DMEM with 2% horse serum. The second exon of the bovine *INSR* gene sequence was amplified to construct the pCD2.1-circINSR overexpression vector (Geneseed, Guangzhou, China). siRNA oligonucleotides were designed to combine with the back-splice region. The miR-34a mimics was synthesized by RiboBio (Guangzhou, China). The mimics (50 nM), siRNA (50 nM), or vectors (2 μg/mL) were transfected into cells with Lipofectamine 2000 (Thermo Fisher Scientific).

### RNA Preparation, Treatment with RNase R, and Actinomycin D

Total RNA in cells and tissues was extracted with TRIzol reagent (Invitrogen, Carlsbad, CA, USA) and reverse transcribed with PrimeScript RT reagent kit (Takara, Tokyo, Japan). Quantitative real-time PCR for RNA analyses were performed using SYBR green PCR master mix (Takara, Tokyo, Japan). A miR-34a-specific stem-loop primer was used to perform reverse transcription. Based on the sequence of circINSR, convergent and divergent primers were designed to determine the authenticity of the circRNA. The divergent primers designed based on the junction site were used for circINSR quantification. Total RNA (1 μg) was purified after being incubated with RNase R (3 U) (Epicenter, Madison, WI, USA) at 37°C for 15 min. Actinomycin D (MilliporeSigma, Burlington, MA, USA) (2 μg/mL) was added to the medium of cells for testing the half-life of circINSR and linear mRNA.

### RNA-FISH

RNA-FISH probes that specifically recognize the back-splicing junction region of circINSR were designed (RiboBio, Guangzhou, China). Cells were fixed with *in situ* hybridization fixative. After being penetrated by 0.1% Triton X-100, the cells were incubated with the circINSR probes at 37°C overnight. Nuclei were stained with DAPI. Laser confocal microscopy was used to observe the localization of circINSR in cells (Nikon, Tokyo, Japan).

### Dual-Luciferase Reporter Assay

Either the full-length of circINSR or three target-site fragments were inserted into the psiCHECK-2 vector (Promega, Fitchburg, WI, USA). A miR-34a biosensor (psiCHECK-2-miR-34a 2×) was created by inserting two copies of miR-34a reverse complementary sequence in the psiCHECK-2 vector. The predicted 3′ UTR fragment containing the miR-34a binding site in CCNE2 and Bcl-2 was PCR amplified, cloned into the psiCHECK-2 vector, and designated gene-WT. To mutate the presumptive binding sites in *CCNE2* and *Bcl-2*, the binding site sequences were replaced as indicated, and designated gene-MUT.

For the luciferase reporter assays, HEK293T cells were seeded in 96-well plates at 8 × 10^3^ cells per well and co-transfected with 0.2 μg reporter plasmid and 50 nM miR-34a mimic or mimic-NC. The ratio of firefly and renilla luciferase activity was detected with the dual-luciferase reporter assay kit (E2920, Promega, Fitchburg, WI, USA) after 24 h. The optical density of the resulting solution was assessed using the automatic microplate reader (Molecular Devices, Sunnyvale, CA, USA).

### RIP Assay

A RIP assay was conducted using an EZ-Magna RIP kit (17-701, Millipore, Billerica, MA, USA) and an anti-Argonaute2 (anti-Ago2) antibody (Abcam, UK). Approximately 10^7^ cells were collected by cell scraping and resuspended in an equal volume of lysis buffer (50 mL). Magnetic beads were coated with Ago2 antibody and mouse immunoglobulin G (IgG) antibody, respectively. 100 mL of cell lysate was incubated with coated magnetic beads at 4°C overnight. The RNA in the immunoprecipitated product was extracted, and the abundance of circINSR, INSR, and miR-34a was detected by quantitative real-time PCR after reverse transcription.

### Cell Proliferation Assay

Cell proliferation was detected by CCK8 (Multisciences, Hangzhou, China) following the manufacturer’s protocols. The optical density of CCK8 at 450 nm was detected using an automatic microplate reader. We also measured cell proliferation using an EdU assay kit (RiboBio, Guangzhou, China). After the required treatment, EdU (5-ethynyl-2′-deoxyuridine) solution was added to the medium. After a 2-h incubation, proliferating cells were stained with Apollo dye solution. The nuclei were stained with Hoechst 33342. Finally, we used a fluorescence microscope (AMG EVOS, Seattle, WA, USA) to observe cell proliferation.

### Cell Cycle Assay

Myoblasts were transfected with siRNAs, mimics, and plasmids, and the cell cycle was assessed using the cell cycle testing kit (Multisciences, Hangzhou, China). After transfecting for 24 h, cells were harvested and washed with cold phosphate buffered saline (PBS). Then, the staining solution and permeabilization reagent were added to the cells. After incubating for 30 min, the cell cycle was analyzed by flow cytometry (FACS Canto II, BD Biosciences, USA).

### Cell Apoptosis Assay

Cell apoptosis was measured using an annexin V-PE/7-AAD apoptosis detection kit (RiboBio, Guangzhou, China). After transfection, cells were harvested and resuspended in binding buffer. Then, annexin V (5 μL) and 7-AAD (10 μL) were added to each cell suspension and incubation was continued for 10 min. Afterward, the samples were analyzed using flow cytometry. When annexin V-PE is combined with 7-AAD, annexin V-PE can label early apoptotic cells (annexin V^+^/7-AAD^−^), while late apoptotic cells and necrotic cells are simultaneously treated with annexin V-PE and 7-AAD stained double positive (Annexin V^+^/7-AAD^+^).

### ΔΨm Assay

Decreased ΔΨm is a hallmark of early cell apoptosis. The apoptosis of stimulated cells can be evaluated using a mitochondrial membrane potential assay kit with JC-1 (Solarbio, Beijing, China). The cells were stained with JC-1 reagent and then analyzed by flow cytometry. Images were obtained by fluorescence microscope (AMG EVOS, Seattle, WA, USA). After staining with JC-1, the mitochondria of non-apoptotic cells emit orange-red fluorescence. The JC-1 monomers present in apoptotic cells emit green fluorescence. Therefore, cells with increased green fluorescence in the cytoplasm and reduced red fluorescence are considered to have undergone apoptosis.

### Western Blot Analysis

Total proteins of bovine myoblasts were prepared with radio immunoprecipitation assay (RIPA) lysis buffer (Solarbio, Beijing, China). Proteins were fractionated on 12% SDS-PAGE gels and transferred to polyvinylidene difluoride (PVDF) membranes (Thermo Fisher Scientific). Blots were incubated overnight with primary antibodies specific for anti-CyclinD1 (#ab226977), anti-glyceraldehyde-3-phosphate dehydrogenase (GAPDH, #ab9485), anti-MyoD (#ab16148) (Abcam, Cambridge, UK), anti-INSR (#WL02857), anti-PCNA (#WL01804), anti-CDK2 (#WL01543), anti-CyclinE (#WL01072), anti-Bcl-2 (#WL01556), anti-P53 (#WL01919), anti-P21 (#WL0362), anti-MyoG (#WL01132), anti-MyHC (#WL02785), and anti-Bax (#WL01637) (Wanleibio, Haerbin, China) at 4°C. After incubation with secondary antibodies, the membranes were quantified with the ChemiDoc XRS system (Bio-Rad, Hercules, CA, USA).

### Statistical Analyses

Unless otherwise stated, all data are shown as the mean ± standard error of the mean (SEM). Statistical analyses were performed using SPSS 22.0 statistical software (SPSS, Chicago, IL, USA). The differences between two experimental groups was analyzed by Student’s t test. One-way ANOVA was used to compare the statistical significance among more than two groups. The statistical difference was considered significant if p < 0.05 and was indicated with an asterisk, while two asterisks represent a p <0.01.

## Author Contributions

H.C. and X.S. designed research. X.S., W.R., and Y.H. performed experiments and analyzed data. X.S. wrote the paper. C.L. and X.L. contributed new analytic tools. H.C. and X.Z. helped modify the language of this manuscript.

## Conflicts of Interest

The authors declare no competing interests.
